# General practitioners’ experiences with chronic abdominal symptoms and a faecal calprotectin guided referral strategy in children: A Dutch qualitative study

**DOI:** 10.1080/13814788.2024.2432417

**Published:** 2024-12-02

**Authors:** Sophie M. Ansems, Marjolein Y. Berger, Donald G. van Tol, Marijke Olthof, Gea A. Holtman

**Affiliations:** a Department of Primary and Long-term Care, University Medical Center Groningen, University of Groningen, Groningen, the Netherlands; b Department of Sociology, Faculty of Behavioral and Social Sciences, University of Groningen, Groningen, the Netherlands

**Keywords:** Children, faecal calprotectin, qualitative research, functional gastrointestinal disorders, general practitioners, primary care

## Abstract

**Background:**

General practitioners (GPs) often struggle to distinguish functional gastrointestinal disorders (FGID) from organic disorders in children with chronic abdominal symptoms. A referral strategy guided by faecal calprotectin (FCal) testing may help.

**Objective:**

This study explores GPs’ experiences with these children and the strategy.

**Methods:**

GPs were sampled purposively to data saturation. Ultimately, we conducted one focus group session and 13 semi-structured interviews with 17 Dutch GPs who had been involved in a randomised controlled trial evaluating an FCal-testing strategy. The online focus group and interviews were recorded, transcribed verbatim, and subject to thematic content analysis.

**Results:**

Four themes arose: diagnostic confidence, fear of missing something severe, reassurance and managing FGID in primary care. Although GPs typically felt confident during the diagnostic process, they did fear missing somatic or psychosocial conditions. They felt more diagnostically confident due to FCals clear indications, high diagnostic accuracy, and non-invasiveness. Reassurance was considered crucial in children with FGID, either by labelling symptoms, providing explanatory models, or offering medical interventions (e.g. FCal testing). When helping children with FGID proved too difficult, GPs referred to specialist care. Besides the integration of FCal during reassurance, the testing strategy did not help GPs manage children with FGID.

**Conclusion:**

While the FCal-strategy improved diagnosis according to GPs, they found the primary challenge to be managing children with FGID. Nevertheless, they found the FCal-strategy beneficial, likely due to its integration into reassurance strategies. Further research focusing on enhancing communication and interventions for paediatric FGID in primary care is warranted.

## Key Messages

General practitioners (GPs) acknowledged the potential value of faecal calprotectin in the diagnostic evaluation of children with chronic abdominal symptoms.

Faecal calprotectin also supports GPs in reassuring and communicating with children with FGID, but not in relieving their FGID symptoms, while this was the main challenge according to GPs.

## Introduction

Chronic abdominal symptoms are prevalent in children and have major implications on a child’s wellbeing and the wider healthcare system [1]. An average Dutch general practitioner (GP) will see 10 children with chronic abdominal pain each year [2], of whom nine will have a functional gastrointestinal disorder (FGID) [3,4]. GPs use diagnostic testing, especially blood tests [3], in one-third, and refer another 13%–20% to specialists [3,5]. These strategies help to differentiate FGIDs from severe organic disorders, such as inflammatory bowel disease (IBD) and coeliac disease, that have serious consequences if missed [6,7]. By contrast, avoidable testing and referral for FGIDs could lead to medicalisation and increases the burdens on the child and healthcare system [8].

Faecal calprotectin (FCal) is a non-invasive test that can safely exclude IBD and could guide GP decisions [5]. However, it is currently used in <1% of Dutch children with non-acute abdominal symptoms in primary care current Dutch guidelines recommend against its use in primary care due to insufficient evidence [9]. Previous studies in adults show that physicians use diagnostic tests for reasons beyond diagnostic uncertainty, including reassurance, being seen “to be doing something,” and saving time [10–13]. Referrals may also be used to strengthen the doctor-patient relationship or offer legal protection for the physician [14]. Insights into how GPs make management decisions in children with chronic abdominal symptoms could lead to improved care and identify a role for FCal.

This qualitative study explores GPs’ experiences and reasons for management decisions, focusing on FCal testing and referrals in children with chronic abdominal symptoms.

## Methods

### Study design

This qualitative study had a realist approach [15] and used a deductive-inductive method, integrating pre-existing insights from the literature with new insights derived by analysis [16]. With realism as our foundation, we assume that meaning and experience are inherent to individuals, and that language reflects these inner experiences, thereby allowing motivations, experiences, and meanings to be analysed in a direct manner [15]. We conducted a focus group session in June 2021, followed by semi-structured interviews between July 2021 and October 2022. Reporting follows the Consolidated Criteria for Reporting Qualitative Research (COREQ) checklist [17]. The Medical Research Ethics Committee of University Medical Centre Groningen approved this study (Research Register: 20210071).

### Recruitment and selection of respondents

We have conducted a randomised controlled trial (RCT) into whether an FCal-guided referral strategy in primary care could reduce referral rates (results pending) [18]. GPs from the northeast of the Netherlands involved in this RCT were eligible to participate if they consented to be approached for further studies [18]. We used purposive sampling based on gender, GP experience (years), practice location (urban vs. rural), percentage of children of total practice population and study arm. Inclusion continued until data saturation, defined as the moment when interviews yielded no new insights [16]. The first author invited 98 of 127 GPs and 90 of 122 GPs from the intervention and control groups, respectively. All participants received an information letter and provided informed consent.

### The FCal-guided referral strategy

In the RCT, GPs in the intervention group received a 60-minute online training session (Box 1). This specified that they could only test children with alarm symptoms for IBD, basing management decisions on the FCal result: <50 μg/g, do not refer (IBD excluded); 50–250 μg/g, monitor; and >250 μg/g, refer. FCal point-of-care-test devices were made available in their practices. GPs in the control group were asked to follow the Dutch Society of GP guidance not to perform FCal testing. GPs in the control group were informed about the intervention without detailing the FCal strategy.

Box 1. The five modules of the online training about the FCal-guided referral strategy in the intervention group of the RCT

1. **Introduction:** The aim of this module is to teach the GP about the differential diagnosis, prevalence, and definitions of chronic abdominal symptoms in children in primary care.
2. **Case 1: *A teenager in whom there is a high suspicion of IBD.*** This module aims to teach the GP about the alarm symptoms for IBD, the diagnostic value of FCal (cut-off value >250 μg/g) and the causes of false-positive results.
3. **Case 2: *A school-aged child with functional abdominal pain.*** This module aims to teach the GP about the indications for testing (alarm symptoms) and the diagnostic value and the follow-up approach for FCal values between 50 and 250 μg/g.
4. **Case 3: *A teenager with chronic abdominal pain and one alarm symptom.*** This module aims to teach the GP about the diagnostic value of FCal values <50 μg/g and the pros and cons of referral. It also provides tips and explanatory models for the communication with a child and parents about FGID.
5. **Proficiency test:** The test includes 10 questions that address the key messages of the online training. The GP has three chances to attain seven correct answers.

GP: General practitioner; IBD: Inflammatory bowel disease; FCal: Faecal calprotectin.

### Data collection

A topic list was prepared using sensitising concepts [19] from the literature and an *a priori* expert discussion within the research team (1 physician, 2 academic GPs, 1 sociologist, 1 primary care researcher): role of medical interventions, doctor-patient relationship, and explanatory model for symptoms (Appendix 1). The topic list was used for the focus group guide (Appendix 2), and the insights derived from the focus group were used to amend the interview guide (Appendix 3).

#### Focus group

To explore GP’s experiences with children with chronic abdominal symptoms and the factors influencing their management decisions seven GPs from the control group took part in a focus group. We chose control group GPs because their experiences had not been influenced by the FCal strategy and knowledge gained from the online training. “Children with chronic abdominal symptoms” were defined as those aged 4–18 years, with chronic abdominal pain and/or diarrhoea, without high suspicion of severe pathology, and consequently, who had a high probability of FGID.

An experienced and independent female moderator (Annemieke Visser, PhD Applied Health Sciences) facilitated the focus group. She had no prior relationship with the participating GPs or previous research. The focus group guide contained open-ended questions about GPs’ experiences, management decisions (diagnostic testing, starting (non-)medical intervention, active follow-up, referring to hospital or paramedic caregivers) and communication (Appendix 2). SMA and GAH observed the focus group, paying attention to nonverbal communication, group dynamics, and topic coverage.

The focus group lasted 2 h, was audio- and video-recorded, and transcribed verbatim by SMA. A written synopsis of the focus group session was discussed within the research team.

#### Interviews

To allow deeper exploration in a less socially influenced setting, the same moderator interviewed different GPs from both arms of the RCT As a result of new insights derived from the focus group, the interviews narrowed their focus on communication- and reassurance strategies and the role of diagnostic testing and referrals (Appendix 3). With participants from the intervention group, the moderator discussed the impact of the FCal-strategy on their management decisions. After five interviews, it became clear that having a deeper medical knowledge would enhance the discussions, so the first author (SMA, a female doctor and trained qualitative interviewer) conducted five additional interviews and repeated three. SMA had met all participating GPs at least once during the RCT.

The online interviews lasted 17–42 min, were audio- and video-recorded, and then transcribed verbatim by an independent transcription service. All GPs received an interview synopsis for member checking. Quotes were translated from Dutch to English by SMA, edited by a native English speaker, and then rechecked by SMA to ensure their meaning remained.

### Data analysis

Data collection and thematic content analysis [15] were performed concurrently, allowing emerging insights and (candidate) themes to be incorporated and explored in subsequent interviews. The focus group session and first three interviews were coded in a data-driven manner [15] by SMA and GAH separately for standardisation, discussing and resolving inconsistencies by consensus to develop an initial coding framework. SMA then applied the coding framework to further transcripts, which GAH verified. The coding framework underwent continuous revision until the eighth interview, at which point the final coding framework was established and candidate themes were identified [15]: the feeling of diagnostic and/or therapeutic (in) competence, fear of missing something severe, the GP-patient-relationship, GP factors, child/parent factors and faecal calprotectin. Through reviewing the candidate themes, final themes were defined and shared with respondents, asking whether they agreed or disagreed with them and providing an opportunity for feedback. When quoted, participants were also asked if quotes retained their intended meaning. All analyses were facilitated using Atlas.ti 9.1 software.

Notes about the (candidate) themes and their relationships were made throughout the analysis, and provisional findings were discussed critically in research team meetings.

## Results

### Respondents

Seventeen GPs took part (Table 1). Data saturation appeared after one focus group, eight interviews, and three repeat interviews. To ensure saturation we continued purposive recruitment and interviewed one more GP each from both study arms. All GPs agreed with the first member check (interview synopsis). During the second member check (results), two GPs agreed after minor adjustments, four agreed without any changes, and eleven did not respond (Appendix 4).

**Table 1.** GP characteristics (*n* = 17).

Characteristic	Number
Method of data collection	
Focus group	7
Interview	10
Repeat-interview	3
Study arm, intervention	7
FCal POCT available in practice during trial	7
Gender, male	8
GP work experience, years	
0–9	7
10–19	6
20–29	2
30–35	2
GP type	
Practice owner	13
GP locum	4
Location GP practice, urban^a^	7
Percentage of children in practice	
<20%	5
20–24%	9
≥25%	3

FCal: Faecal calprotectin; POCT: Point-of-care testing; GP: General practitioner.

^a^Rural area is defined as <500 addresses/km^2^ [20].

We identified four main themes that GPs face when managing a child with chronic abdominal symptoms: feeling confident in the diagnostic process, fear of missing something severe, reassurance and feeling able to manage FGID in primary care.

### Themes

#### Feeling confident in the diagnostic process

Most GPs felt certain about the diagnostic process in most children with chronic abdominal symptoms, knowing that most have FGIDs and do not require diagnostic testing to exclude a serious organic illness. They might use other tools, including growth monitoring or watchful waiting.

“But I often don’t feel insecure. Actually, when I see the child, I often have a sense of “this is fine.” And, if you monitor them, you can see how things develop. No, I’m not worried about missing something.” (GP 5, control group)

GPs mentioned wanting to protect children from invasive diagnostic testing, especially blood tests, which they reserved for cases with a clear objective indication, such as alarm symptoms.

Most intervention group GPs appreciated the non-invasive nature of FCal, but a few preferred that blood tests could also exclude coeliac disease. Nevertheless, the FCal-guided referral strategy led to most GPs feeling more secure in their diagnoses. The online training helped by refreshing their knowledge about alarm symptoms and the very low probability of serious organic disease.

GP 11 (intervention group): *“The part that I used to let the pediatrician handle … I can now just do myself with sufficient certainty.”*

Interviewer: *“What do you mean by ‘doing it yourself?’ Do you mean calprotectin?”*

GP 11 (intervention group): *“Yes, but also the history; with growth and abdominal pain, how to ask about that and when to think about something unusual or not.”*

One GP did not see the added value of FCal in the diagnostic pathway, stating that he felt confident using watchful waiting or immediate referral in case of high suspicion of organic illness.

#### Fear of missing something severe

While most GPs felt diagnostically confident in most cases, GPs also expressed to fear missing a severe diagnosis in some cases. For some, this was stronger when meeting a child for the first time (e.g. a locum GP and a GP working in an out-of-hours setting). Parental pressure on the GP to ensure that the GP did not miss anything also influenced this feeling.

“Even if you are convinced that it is something very innocent (…) you can never completely rule out that something serious is going on.” (GP 17, control group)

Children without clear signs of serious physical illness or psychosocial influences on their symptoms were labelled as being in a “gray area” and were considered a difficult group to diagnose. One GP admitted feeling relieved when a child had alarm symptoms because it removed the ambiguity. GPs sometimes referred children in this gray area early, arguing that paediatricians were better placed to determine appropriate diagnostic testing.

“For cases that are clearly abnormal, you think, ‘I don’t know what it is, but it’s not right.’ … you refer those. I do that more quickly than in adults.” (GP 3, control group)

Other GPs were concerned about missing chronic abdominal symptoms as a manifestation of severe psychosocial issues, such as child abuse or bullying, noting that this was hard to recognise.

“I’m always afraid that a child will come back as an adult and say, ‘Doctor, back then I came to you, but the abuse just continued,’ or whatever; that’s my biggest fear.” (GP 6, control group)

Most intervention group GPs believed that implementing the FCal-strategy had prevented referrals to specialist care, particularly by increasing their confidence about children in the gray area. The clear indications for FCal testing and its high reliability for excluding IBD contributed to this confidence.

“But there are cases where you do think: ‘This is unusual or different,’ and then I do find it comforting … I dare to just say, with a negative calprotectin, that we’ve also ruled that out.” (GP 10, intervention group)

#### Reassurance

GPs emphasised the importance of reassurance in children with FGIDs. They employed various methods to provide reassurance, one of which was offering a clear explanation of the nature of the symptoms—an ‘explanatory model’.

Some GPs felt they should believe this model themselves, irrespective of the scientific basis.

“I never try to state something I don’t believe myself. […] I truly believe that’s how it works: things are expressed through symptoms and how they influence each other. I have a little model for that, and I believe in it. Whether it’s completely accurate and well-founded may be questionable, but I believe in it.” (GP 6, control group)

Others preferred to view explanatory models as communication techniques or skills, without needing to believe it themselves.

“Providing the framework of an irritable bowel and then hanging everything on it, that’s a bit of a technique for me. I don’t use that when my own children have functional abdominal pain. It’s really a way to give people something to hold on to that doesn’t rely on the truth.” (GP 2, control group)

Although GPs saw reassurance and patient education as core tasks, they considered it difficult, especially when the GP-patient relationship is weak or psychosocial factors are absent.

“Functional abdominal complaints aren’t necessarily easy to treat … people often don’t understand how you can have abdominal pain when there’s nothing really wrong […] If the psychosocial causes are obvious, maybe it’s easier […] But if they are less prominent, then it sometimes difficult for people to accept.” (GP 12, intervention group)

A strong GP-patient relationship facilitated reception of the explanatory model by parents and children.

“I have a pretty good relationship with my patients. So, I do have the trust of most. That’s why they’re willing to accept my advice. Many parents just want to be reassured and receive confirmation that it’s probably related to stress. They just go to the doctor for peace of mind.” (GP 13, intervention group)

GPs used diagnostic testing for reassurance. This could follow a parental request for a specific test (e.g. coeliac disease) or could be used deliberately to achieve reassurance.

GP 14 (control group): *“Possibly an abdominal ultrasound, it’s not so burdensome and also has a magical influence.”*

Interviewer: “*But you say: ‘magical influence.’ So that’s more.”*

GP 14 (control group): “*… for reassurance."*

One GP mentioned that knowing the child and parents well helps them assess whether a negative test result would be reassuring. However, other GPs emphasised that diagnostic testing could cause more parental concern and child distress. One GP stated that testing was mostly done under parental pressure, not to improve the child’s wellbeing.

“And the uncomfortable part is that you’re usually dealing with the wishes of parents rather than just the children. Because if it were just about the children, you’d almost never have to perform diagnostics.” (GP 10, intervention group)

FCal was also used for reassurance. GPs expressed that if they believed the child was not suffering from a serious underlying disease, confirmed by a negative FCal test, this radiated from them and facilitated reassurance. Although GPs were aware of the downsides of frequent FCal testing, they tested children without suspected IBD and used a negative FCal value in their explanation of FGID.

“As much as I dislike it, I do it anyway. I am aware that I do it more than necessary, but it helps to say, ‘Look, this is low, ruling out chronic bowel disease.’ I’m talking about the children where you would otherwise have tried to explain without lab results… now you’re going to use that calprotectin. And you feel like you have something in your hands that you can say: ‘Look’.” (GP 10, intervention group)

The availability of FCal point-of-care-testing was thought to facilitate “over-testing,” because the perceived testing threshold was lower than for standard laboratory tests. Although some GPs did feel that point-of-care-testing allowed for rapid reassurance, others stated that a quick test result was not needed in the context of long-lasting symptoms.

Reassurance was also achieved by referral to specialist care. Some GPs admitted using the “authority” of the paediatrician to convince parents of a functional diagnosis.

“When they hear it from the specialist, they’re more willing to go along with it … a pediatrician stands above us as GPs. You can’t go beyond the pediatrician; so, for some people, that’s kind of the end of the road.” (GP 16, intervention group)

GPs mentioned including a statement in their referral letter mentioning that it was the parents who were concerned about an organic illness. When questioned about the potential detrimental effects of these referrals, GPs noted that paediatricians share a holistic approach and often returned children to their care. The GPs considered these referrals safe, but they did not consider the associated costs.

#### Feeling able to manage FGID in primary care

Many GPs believed that offering reassurance and labelling symptoms (e.g. “irritable bowel syndrome”) usually sufficed and allowed parents and children to deal with the symptoms without further consultations.

[about labeling] GP 3 (control group): *“That is also more efficient; then you can wrap it up … you can move forward.”*

GP 4 (control group): “*Yeah, and what [GP 6] just said … it works well. I see those children very infrequently after. You’ve given them something to work with … of course, they still have their abdominal pain. If they come for something else, and you ask about it, they still have it occasionally, but it’s not a problem they come back with often.”*

When asked whether the lack of consultations implies the absence of symptoms, GPs highlighted the inherent limitations of their role, emphasising that they cannot resolve every issue. If symptoms persisted, however, GPs stated that they primarily offered guidance instead of immediate treatment. Sometimes starting a specific treatment, such as prescribing laxatives, helped them “take control.” In general, GPs stated that children with FGIDs belong in primary care. Some GPs felt more competent about managing FGIDs than paediatricians because of their familiarity with the child, their family, and other primary care providers (e.g. physical therapists and youth physician assistants).

“Because I have more information about the family background, dynamics, debt issues (…) and shorter lines of communication with relevant organizations (…) I have more tools to be able to do something.” (GP 13, intervention group)

By contrast, locum or inexperienced GPs were unaware of referral options within their network so referred to the paediatrician quicker. GPs would also refer to a specialist if symptoms persisted or had a severe impact on the child’s daily life, because they believed paediatricians have more expertise in the treatment of FGID and have access to centralised care (e.g. *poep- en plaspoli*; a poop and pee outpatient clinic for children) or specialised treatments, such as hypnotherapy.

“I’m aware that there are excellent clinics … and sometimes it gives parents peace of mind … their child has been examined by a specialist who has looked into things more thoroughly, and they often accept it more easily. […] Additionally, a pediatrician has access to psychologists and hypnotherapy, which [are] effective.” (GP 8, intervention group)

The FCal-strategy had a limited impact on managing children with FGID in primary care. Most GPs in the intervention group were already familiar with the explanatory models for FGID described in the online training (e.g. because it was covered during GP training). Some GPs in the intervention group did express a desire to know more about the treatment options for FGID, which was missing in the online training.

[about the online training] *“…tips and tricks for communication. I think, ‘yes, that makes sense.’ It’s also practical. But perhaps the next step … Well, suppose someone has functional abdominal pain. What next?”* (GP 8, intervention group)

### The role of FCal testing in daily practice

**Figure 1.**
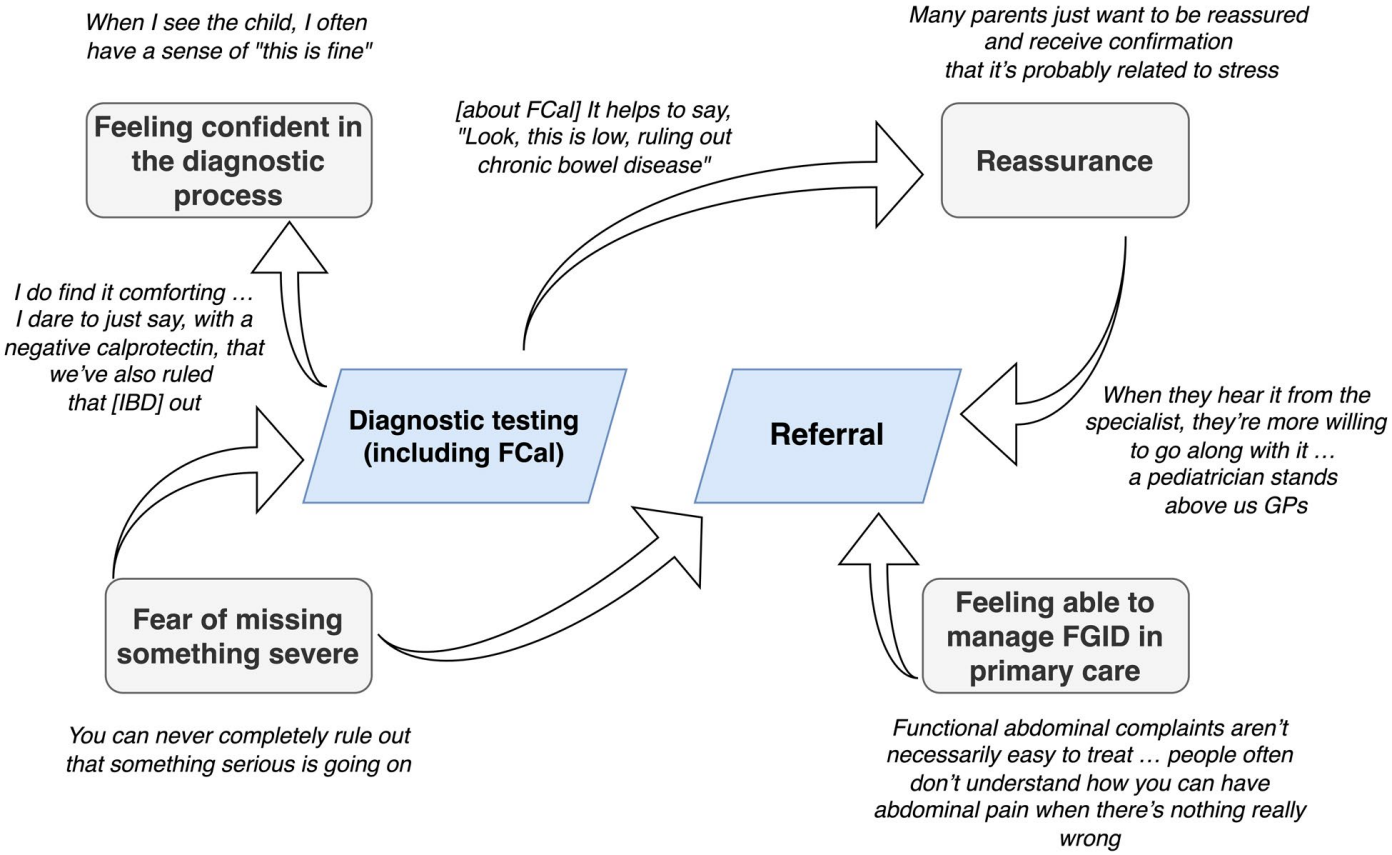
Visualisation of the four themes (grey rectangles) and their influence on diagnostic testing (including FCal) and referral according to the participating GPs. Participant quotes are displayed in italics. When GPs feared missing something severe, they sometimes used (FCal) testing to feel more confident in the diagnostic process. In addition, FCal testing was used to reassure parents and children. When GPs felt unable to reassure parents or manage FGID in primary care, they referred children to specialist paediatric care. Finally, GPs sometimes referred children to paediatric care when they feared missing something severe. IBD: Inflammatory bowel disease; GP: General practitioner; FCal: Faecal calprotectin; FGID: Functional gastrointestinal disorder.

The four themes and how they influence diagnostic testing and referral according to the participating GPs are summarised in Figure 1.

## Discussion

### Main findings

While GPs expressed usually feeling confident about diagnosing children with chronic abdominal symptoms, they also mentioned fearing missing a severe somatic or psychosocial condition in certain circumstances. The clear indications, high diagnostic accuracy, and non-invasiveness of the FCal-guided referral strategy helped GPs in the diagnostic process. Managing FGIDs was generally considered demanding and relied on effective reassurance of children and parents through symptom labelling, explanatory models, diagnostic testing, and specialist referral. Some GPs preferred to refer children with persistent symptoms to specialist care. Besides its integration in reassurance strategies, the FCal-guided referral strategy had limited impact on actually managing children with functional gastrointestinal disorders.

### Strengths and limitations

First, although the transitions from focus group to interviews and to another interviewer were both unplanned, we believe this approach improved the credibility of our findings [21]. The initial interviewer had no prior involvement in the project and could encourage open discussions about the general experiences of GPs. Transitioning to a different interviewer with a medical background and pre-existing relationship with the GPs benefitted from a shared understanding of the pros and cons of diagnostic tests and referrals. The two member checks, frequent debriefing sessions and thick description of our findings also contribute to the credibility of this study [22]. Second, although the research team consisted of researchers with and without involvement in the RCT evaluating the FCal-strategy, only one research team member had a non-medical background (DvT, sociologist). This could have led to a predominantly medical perspective. Third, only 7 of the 17 GPs were from the intervention group; however, this issue was somewhat mitigated by repeating three interviews in that group. Fourth, despite being a diverse group, we only included GPs who participated in a trial about FCal in children with chronic abdominal symptoms. Consequently, participating GPs may have been more academically oriented and may have had greater affinity with this patient group than other Dutch GPs. The findings of our study must be understood within the context of our sample of GPs working in Dutch primary health care in 2021–2022. Lastly, our attempts to provide an in-depth description of our methodology and data-analysis contribute to the dependability and confirmability of our findings [22].

### Comparison to existing literature

As hypothesised in the introduction [10–13], GPs used tests (e.g. FCal) for more than reducing diagnostic uncertainty. This helps explain the high rate of diagnostic testing in children with non-acute abdominal symptoms reported in an earlier retrospective cohort study [3]. GPs expressed to use FCal-testing for its high negative predictive value, hoping a negative result would help them reassure parents and prevent referral. However, they rarely mentioned the potential for (false-)positive results and possible downstream consequences. GPs particularly appreciated the added value of FCal for children in the so-called gray area. The study only provides some clues as to why children end up in this area, with GPs mentioning they felt more insecure when they did not know the child well, when pressured by parents, and when no clear psychosocial or alarming symptoms were present. Indeed, most children who present to primary care are still in the early stage of the disease, before alarm symptoms develop [23]. At first, it seems paradoxical that GPs can feel confident in their diagnostic process while simultaneously fearing missing something severe. However, we emphasise that diagnosis in primary care is often characterised by uncertainty [24]. Although most GPs had positive experiences with the FCal-guided referral strategy, conclusive decisions about its implementation will depend on the upcoming RCT findings and its patient outcomes (e.g. referral and parental concern) [18].

Most GPs emphasised the importance of an explanatory model to reassure parents and children, but they could find this challenging, especially in the absence of a clear psychosocial cause or when they did not believe in the model themselves. This finding aligns with previous studies in patients with persistent somatic symptoms [25,26]. A solution might be to develop a clear evidence-based communication strategy for GPs that can be delivered with a patient information leaflet [27].

GPs reported integrating a negative FCal value into explanations about FGID, even when they did not suspect IBD before testing. However, although such an approach may have utility with a subset of patients, there is no evidence that negative test results provide reassurance to patients [28]. Instead, parents and children want support, understanding, and an explanation, and the lack of planned follow-up can leave them feeling isolated [29]. Notably, most GPs relied on self-management without further consultation. GPs may underestimate the effect of taking time for an adequate explanation in children with non-acute abdominal symptoms, as underpinned by the low follow-up rate [3] and frequent persistence of symptoms [30].

GPs sometimes felt that they lacked the tools to treat children with FGID, often leading to specialist referrals. This conflicts with a similar study by Brodwall et al. [31], who found that Norwegian GPs felt comfortable serving as the primary care provider for children and adolescents with FGIDs and seldom referred these patients [31]. A possible explanation could be that Norwegian GPs have more time per contact [32]. The Dutch Society of GPs advocates patient education and a balanced diet, but discourages all other (non-) medical treatments [9]; however, this approach denies the fact that symptoms often persist [30]. Dutch and international secondary care guidelines recommend using cognitive behavioural therapy and hypnotherapy in reducing FGID symptoms [33,34]. However, there is no high-quality evidence showing the effectiveness of cognitive behavioural therapy nor hypnotherapy for children with FGIDs in primary care.

## Conclusion

The FCal-strategy made GPs feel confident in their diagnosis and helped them in reassuring and communicating with children with FGID. However, the impact of the FCal-strategy on managing children with FGID was limited, while this was the primary challenge according to GPs. Primary care interventions to improve the management of children with chronic abdominal symptoms should therefore focus on effective communication [27] and strategies that can relieve symptoms in this setting. Such advances could empower GPs to treat such children in primary care.

## Supplementary Material

Supplemental Material

## References

[1] Chitkara DK, Rawat DJ, Talley NJ. The epidemiology of childhood recurrent abdominal pain in western countries: a systematic review. Am J Gastroenterol. 2005;100(8):1868–1875. doi: 10.1111/j.1572-0241.2005.41893.x. 16086724

[2] Gieteling MJ, Lisman-van Leeuwen Y, van der Wouden JC, et al. Childhood nonspecific abdominal pain in family practice: incidence, associated factors, and management. Ann Fam Med. 2011;9(4):337–343. doi: 10.1370/afm.1268. 21747105 PMC3133581

[3] Ansems SM, Berger MY, Pieterse E, et al. Management of children with non-acute abdominal pain and diarrhea in Dutch primary care: a retrospective cohort study based on a routine primary care database (AHON). Scand J Prim Health Care. 2023;41(3):267–275. doi: 10.1080/02813432.2023.2231054. 37427876 PMC10478593

[4] Hyams JS, Lorenzo CD, Saps M, et al. Childhood functional gastrointestinal disorders: child/adolescent. Gastroenterol. 2016;150(6):1456–1468.e2. 10.1053/j.gastro.2005.08.063 PMC7104693 16678566

[5] Holtman GA, Lisman-van Leeuwen Y, Kollen BJ, et al. Diagnostic accuracy of fecal calprotectin for pediatric inflammatory bowel disease in primary care: a prospective cohort study. Ann Fam Med. 2016;14(5):437–445. doi: 10.1370/afm.1949. 27621160 PMC5394359

[6] Ricciuto A, Fish JR, Tomalty DE, et al. Diagnostic delay in Canadian children with inflammatory bowel disease is more common in Crohn’s disease and associated with decreased height. Arch Dis Child. 2018;103(4):319–326. doi: 10.1136/archdischild-2017-313060. 28794097

[7] Adler J, Lin CC, Gadepalli SK, et al. Association between steroid-sparing therapy and the risk of perianal fistulizing complications among young patients with crohn disease. JAMA Netw Open. 2020;3(6):e207378. doi: 10.1001/jamanetworkopen.2020.7378. 32515798 PMC7284306

[8] Lindley KJ, Glaser D, Milla PJ. Consumerism in healthcare can be detrimental to child health: lessons from children with functional abdominal pain. Arch Dis Child. 2005;90(4):335–337. doi: 10.1136/adc.2003.032524. 15781917 PMC1720365

[9] Gieteling MJ, van Dijk PA, de Jonge AH, et al. NHG-Standaard Buikpijn bij kinderen. Huisarts Wet. 2012;55(9):404–409.

[10] Watson J, Whiting PF, Salisbury C, et al. Blood tests in primary care: a qualitative study of communication and decision-making between doctors and patients. Health Expect. 2022;25(5):2453–2461. doi: 10.1111/hex.13564. 35854666 PMC9615068

[11] Michiels-Corsten M, Donner-Banzhoff N. Beyond accuracy: hidden motives in diagnostic testing. Fam Pract. 2018;35(2):222–227. doi: 10.1093/fampra/cmx089. 28973135

[12] van der Weijden T, van Bokhoven MA, Dinant G-J, et al. Understanding laboratory testing in diagnostic uncertainty: a qualitative study in general practice. Br J Gen Pract. 2002;52(485):974–980. 12528582 PMC1314466

[13] Lam JH, Pickles K, Stanaway FF, et al. Why clinicians overtest: development of a thematic framework. BMC Health Serv Res. 2020;20(1):1011. doi: 10.1186/s12913-020-05844-9. 33148242 PMC7643462

[14] Tzartzas K, Oberhauser PN, Marion-Veyron R, et al. General practitioners referring patients to specialists in tertiary healthcare: a qualitative study. BMC Fam Pract. 2019;20(1):165. doi: 10.1186/s12875-019-1053-1. 31787078 PMC6885318

[15] Braun V, Clarke V. Using thematic analysis in psychology. Qual Res Psychol. 2006;3(2):77–101. doi: 10.1191/1478088706qp063oa.

[16] Hennink M, Hutter I, Bailey A. Qualitative research methods. Los Angeles, CA: SAGE Publications Sage CA; 2020.

[17] Tong A, Sainsbury P, Craig J. Consolidated criteria for reporting qualitative research (COREQ): a 32-item checklist for interviews and focus groups. Int J Qual Health Care. 2007;19(6):349–357. doi: 10.1093/intqhc/mzm042. 17872937

[18] Ansems S, Berger M, Rheenen P V, et al. Effect of faecal calprotectin testing on referrals for children with chronic gastrointestinal symptoms in primary care: study protocol for a cluster randomised controlled trial. BMJ Open. 2021;11(7):e045444. doi: 10.1136/bmjopen-2020-045444. PMC8311316 34301652

[19] Bowen AG. Sensitizing concepts. London: SAGE Publications Ltd. 2019.

[20] Centraal Bureau voor Statistiek (CBS). Gebieden in Nederland [Areas in The Netherlands]. Omgevingsadressendichtheid. 2022. https://www.cbs.nl/nl-nl/onze-diensten/methoden/begrippen/omgevingsadressendichtheid-van-een-adres

[21] Fusch P, Fusch GE, Ness LR. Denzin’s paradigm shift: revisiting triangulation in qualitative research. J Sustain Soc Chang. 2018;10(1):2.

[22] Shenton AK. Strategies for ensuring trustworthiness in qualitative research projects. EFI. 2004;22(2):63–75. doi: 10.3233/EFI-2004-22201.

[23] McCowan C, Fahey T. Diagnosis and diagnostic testing in primary care. Br J Gen Pract. 2006;56(526):323–324. 16638245 PMC1837838

[24] Malterud K, Guassora AD, Reventlow S, et al. Embracing uncertainty to advance diagnosis in general practice. Br J Gen Pract. 2017;67(659):244–245. doi: 10.3399/bjgp17X690941. 28546389 PMC5442924

[25] Olde Hartman TC, Hassink-Franke LJ, Lucassen PL, et al. Explanation and relations. How do general practitioners deal with patients with persistent medically unexplained symptoms: a focus group study. BMC Fam Pract. 2009;10(1):68. doi: 10.1186/1471-2296-10-68. 19775481 PMC2758831

[26] Houwen J, Lucassen PLBJ, Verwiel A, et al. Which difficulties do GPs experience in consultations with patients with unexplained symptoms: a qualitative study. BMC Fam Pract. 2019;20(1):180. doi: 10.1186/s12875-019-1049-x. 31884966 PMC6935475

[27] Hall-Patch L, Brown R, House A, et al. Acceptability and effectiveness of a strategy for the communication of the diagnosis of psychogenic nonepileptic seizures. Epilepsia. 2010;51(1):70–78. doi: 10.1111/j.1528-1167.2009.02099.x. 19453708

[28] Rolfe A, Burton C. Reassurance after diagnostic testing with a low pretest probability of serious disease: ­systematic review and meta-analysis. JAMA Intern Med. 2013;173(6):407–416. doi: 10.1001/jamainternmed.2013.2762. 23440131

[29] Ansems SM, Ganzevoort IN, van Tol DG, et al. Qualitative study evaluating the expectations and experiences of Dutch parents of children with chronic gastrointestinal symptoms visiting their general practitioner. BMJ Open. 2023;13(5):e069429. doi: 10.1136/bmjopen-2022-069429. PMC10193100 37192810

[30] Lisman-van Leeuwen Y, Spee LAA, Benninga MA, et al. Prognosis of abdominal pain in children in primary care-A prospective cohort study. Ann Fam Med. 2013;11(3):238–244. doi: 10.1370/afm.1490. 23690323 PMC3659140

[31] Brodwall A, Brekke M. General practitioners’ experiences with children and adolescents with functional gastro-intestinal disorders: a qualitative study in Norway. Scand J Prim Health Care. 2021;39(4):543–551. doi: 10.1080/02813432.2021.2012347. 34930079 PMC8725859

[32] Beer L, Cohidon C, Senn N. General practitioner time availability per inhabitant per year: a new indicator to measure access to primary care. Front Heal Serv. 2022;2:832116. 10.3389/frhs.2022.832116 PMC10012817 36925785

[33] Tabbers MM, Benninga MA, Venmans LMAJ, et al. Guideline: Functionele buikpijn bij kinderen [Funtional abdominal pain in children]. Nederlandse Vereniging voor Kindergeneeskunde [Dutch Society of Pediatrics]. 2021.

[34] Gordon M, Sinopoulou V, Tabbers M, et al. Psychosocial interventions for the treatment of functional abdominal pain disorders in children: a systematic review and meta-analysis. JAMA Pediatr. 2022;176(6):560–568. doi: 10.1001/jamapediatrics.2022.0313. 35404394 PMC9002716

