# Supplementary material for: General practitioners’ experiences with chronic abdominal symptoms and a faecal calprotectin guided referral strategy in children: A Dutch qualitative study
Source: Eur J Gen Pract. 2024 Dec 2;30(1):2432417. doi: 10.1080/13814788.2024.2432417 (PMC11613343; doi:10.1080/13814788.2024.2432417)
Supplement: Supplemental Material [file IGEN_A_2432417_SM7300.pdf]

Appendix 1. Topic list

General experiences with pediatric functional gastrointestinal disorders
(Unpublished survey among 50 GPs, performed by a medical student from the Department of Primary and Long-Term Care, University Medical Center Groningen, The Netherlands)

- A majority of GPs agreed (a little) with the statement 'A child with functional abdominal pain is time-consuming'.
- Majority of GPs (63%) indicated that the intervention and not the diagnosis is the most time-consuming.
- Twenty GPs (41%) felt more or less uncertain during their handling of a child with functional abdominal pain. Over one quarter of GPs had, to a greater or lesser extent, the feeling to stand empty handed regarding a child with functional abdominal pain. Also over one quarter of GPs (n=14, 29%) felt that there are insufficient effective treatment strategies available for a child with functional abdominal pain in primary care. Seven GPs (14%) (slightly) disagreed with the statement 'Intervention in children with functional abdominal pain produces good results'.

General experiences with medically unexplained symptoms (MUS)

- Several qualitative studies show that physicians often have negative feelings towards patients with Medically Unexplained Symptoms (MUS), such as feelings of powerlessness and frustration. (1–3)

Structuring the consultation and the doctor-patient relationship

- Interview study among general practitioners: GPs indicated that patients with Medically Unexplained Symptoms (MUS) dominated consultations and directed the conversation. This felt uncomfortable for the physicians. (2)
- General practitioners structure consultations about MUS less effectively and perceive this as a problem. (4,5)
- Multiple physicians emphasize the important of a good doctor-patient relationship, but also express experiencing difficulties while maintaining this relationship. (4,6)
- Parents and children often appreciate clear communication with the child and when the consultation is focused on the child. Parents and children do not like it when the child is not involved in the conversation. (7)

Medical interventions

- Doctors often feel that patients with MUS exert pressure on them and request various medical interventions. (8)
- Doctors feel more inadequate in dealing with MUS than with chronic somatic diseases and often experience (diagnostic) uncertainty, feeling that they should not show this uncertainty to these patients. (1)
- Consultation videos of MUS patients: Half of the patients suggested a somatic intervention (additional tests, medication, referral). MUS patients raised each type of somatic intervention less frequently and during fewer consultations than their GPs. This confirms the suggestion from previous qualitative studies that the initiative for somatic interventions mainly comes from the GPs rather than the patients. (8)
- Interview study among general practitioners: Although GPs were aware that they could help patients by linking psychological factors to somatic symptoms, they often felt incapable of doing so. (2)

- Patients with MUS: Both the doctor and the patient often have the belief that effective treatment options are lacking. (3)

Explanatory model

- Doctors feel more inadequate in dealing with MUS than with chronic somatic diseases and often experience (diagnostic) uncertainty, feeling that they should not show this uncertainty to these patients. (1)
- Doctors spend little time on explanations and often have a very limited arsenal of explanatory models for MUS. (5)
- Although multiple explanatory models are available in the literature, many general practitioners still struggle to explain MUS to patients. Video consultations: GPs often provide brief and undetailed explanations to patients. They communicated the explanatory model as a possibility, and the models were patient-specific. The GPs hardly used extensive explanatory models based on the literature.(9)
- Consultation videos of MUS patients: During this study, it was found that half of the GPS 'normalized' the symptoms, meaning that they indicated that there was no serious condition.(8)
- MUS patients pose a significant challenge for GPs because they do not fit into the existing biomedical model, which primarily focuses on ruling out somatic conditions. However, excluding relevant somatic conditions does not necessarily lead to the patient's recovery.(10)

References

1. Nederlandse Vereniging voor Kindergeneeskunde [Dutch Society of Pediatrics]. NVK Richtlijn [DSP Guideline]: Somatisch onvoldoende verklaarde lichamelijke klachten (SOLK) bij kinderen [Medically Unexplained Symptoms in Children]. 2015

2. Wileman L. Medically unexplained symptoms and the problem of power in the primary care consultation: a qualitative study. Fam Pract. 2002 Apr 1;19(2):178–82.

3. Aiarzaguena JM, Grandes G, Gaminde I, Salazar A, Sánchez Á, Ariño J. A randomized controlled clinical trial of a psychosocial and communication intervention carried out by GPs for patients with medically unexplained symptoms. Psychol Med. 2007 Feb;37(2):283–94.

4. Houwen J, Lucassen PLBJ, Verwiel A, Stappers HW, Assendelft WJJ, Olde Hartman TC, et al. Which difficulties do GPs experience in consultations with patients with unexplained symptoms: A qualitative study. BMC Fam Pract. 2019 Dec 29;20(1).

5. Olde Hartman TC, Hassink-Franke LJ, Lucassen PL, Van Spaendonck KP, Van Weel C. Explanation and relations. How do general practitioners deal with patients with persistent medically unexplained symptoms: A focus group study. BMC Fam Pract. 2009;10:68.

6. Johansen ML, Risor MB. What is the problem with medically unexplained symptoms for GPs? A meta-synthesis of qualitative studies. Vol. 100, Patient Education and Counseling. Elsevier Ireland Ltd; 2017. p. 647–54.

7. Ansems SM, Ganzevoort IN, Van Tol DG, Fokkema T, Olthof M, Berger MY, et al. Qualitative study evaluating the expectations and experiences of Dutch parents of children with chronic gastrointestinal symptoms visiting their general practitioner. BMJ Open. 2023;13.

8. Ring A, Dowrick CF, Humphris GM, Davies J, Salmon P. The somatising effect of clinical consultation: What patients and doctors say and do not say when patients present medically unexplained physical symptoms. In: Social Science and Medicine. Pergamon; 2005. p. 1505–15.

9. Terpstra T, Gol JM, Lucassen PLBJ, Houwen J, van Dulmen S, Berger MY, et al. Explanations for medically unexplained symptoms: a qualitative study on GPs in daily practice consultations. Fam Pract. 2019 Aug 8;37(1):124–30.

10. Rosendal M, Olesen F, Fink P. Management of medically unexplained symptoms. Vol. 330, British Medical Journal. BMJ Publishing Group; 2005. p. 4–5.

Appendix 2. FOCUS GROUP GUIDE – control group

Research questions

1. *What are GPs' experiences of a consultation with a child with chronic GI symptoms?*
2. *What are influencing factors in their management decisions (including referral)?*

Introduction (by Sophie Ansems)

In your practice, you frequently encounter children experiencing prolonged abdominal pain and persistent diarrhea. The DOK 2.0 study explores whether we can enhance primary care by incorporating fecal calprotectin into the diagnostic process. However, this is just a small part of the care pathway for children with chronic abdominal issues. There are other factors influencing the policy decisions of general practitioners. These might serve as opportunities to improve primary care for these children. Through this research, we aim to identify these factors.

The focus of discussion today revolves around the patient group where there's minimal suspicion of pathology, indicating a high likelihood of a functional condition. The initial part of the focus group will center on your general experiences with these children and how you structure your approach. Following the break, we'd like to discuss improving primary care and communication with this patient group.

Annemieke Visser will lead the focus group. Gea Holtman and I will observe and occasionally ask questions. I'll now hand over to Gea for a brief introduction.

Gea Holtman introduces herself → hands over to Annemieke Visser.

Annemieke Visser

- Introduces herself
- Lets GPs introduce themselves
- Communications before we start (free discussion, possible to make comments via the Microsoft Teams chat)
- Audio and video recording of the focus group

START RECORDING

I What is your general experience with children with chronic abdominal complaints (without a high suspicion of organic disease) in general practice?

- Explore and define patient group
    - How often do you see these patients?
    - With what symptoms do they present themselves?
    - What do you generally do during the consultation?
    - What do you think is the most common cause of the symptoms?
    - Is it easy to diagnose these children or do you experience uncertainty?
    - Do they often return for re-consultations?
- What kind of feelings do you experience during the consultation?
    - *Frustration / helplessness*

II What is your usual management with children with chronic abdominal complaints? What does influence your management decisions?

- What are the reasons to either conduct or refrain from diagnostic testing?
    - *Diagnostic uncertainty / parental request / 'Wanting to do something'*
- What are the reasons to prescribe or not prescribe medication?
    - *Parental request / 'wanting to do something'*
- What are the reasons to refer or not refer to a medical specialist?
    - *Parental request / diagnostic uncertainty / assistance in treating functional gastrointestinal disease / 'wanting to do something'*
- What are the reasons to initiate a non-medical treatment?
    - *E.g. Nutrition, stress management, referral to other primary care provider*
- How do you monitor these children?
    - *Do you plan follow-up appointments? Why or why not?*

BREAK if needed (dependent of flow of discussion, around 17:00)

III What would help you to improve the management for these children?

- Further develop diagnostic options?
- More knowledge/expertise?
- Referral options?

IV How do you experience the communication with these patients?

- What do you find easy?
- What do you find difficult? *(unstructured consultation / parents dominate the conversation)*

V Communication – Explanatory model functional gastrointestinal symptoms

- Can you easily explain the symptoms? How do you explain the symptoms?
    - *'Severe organic disease ruled out / extensive explanatory model / metaphor*
- Do you find it difficult to explain the model to parents and children? If yes, why?
    - *Diagnostic uncertainty / does not fit within biomedical model*

For each theme:

- Is there a difference between child and parent?
- Is this a part of your standard practice or is this different fort his specific patient population? *(influencing factors: experience as GP / GP locum or practice owner / medical knowledge)*

STOP RECORDING

Closure (Sophie)

Explanation about transcription, analysis, following interviews with the intervention group.

Ultimate aim is to publish as a scientific article, which will be send to all participants.

Appendix 3. INTERVIEW GUIDE – intervention group

Research questions

1. *What are GPs' experiences of a consultation with a child with chronic GI symptoms?*
2. *What are influencing factors in their management decisions (including referral)?*
3. *How did the fecal calprotectin guided referral strategy influence their management decisions?*

Introduction of the interviewer

START RECORDING

Introduction

In your practice, you often encounter children with prolonged abdominal pain and persistent diarrhea. For the DOK 2.0 study, you underwent an online training about the fecal calprotectin guided referral strategy and used fecal calprotectin (when indicated) in this group of children. In this interview, I aim to assess how you have experienced this intervention and whether they could be further improved.

The patients I woud like to focus on today comprises children where there is minimal suspicion of pathology, suggesting a high likelihood of a functional condition. The initial segment of the interview will delve into your overall experience with these children, how you formulate your approach, and the role of calprotectin within that context. Afterwards, I'd like to discuss the online training: what insights you gained and how we could potentially enhance it.

Informed consent

Emphasize that the audio and video of this conversation will be recorded. Data will be processed anonymously, assigning a code known only to two researchers. The purpose is a scientific publication, and participants will receive a copy beforehand.

I What is your general experience with children with chronic abdominal complaints (without a high suspicion of organic disease) in general practice?

- Explore and define patient group
  - How often do you see these patients?
  - With what symptoms do they present themselves?
- What are, in your perception, the expectations and needs of parents and child?
  - Rule out serious illness / symptom relief / provide 'medical' label / find explanation
- What is, in your experience, the most common cause of the symptoms?
  - Stress or 'between the ears' / constipation / irritable bowel syndrome / unclear
  - In case of stress: how does your explanation link to this?

II How do you reassure parents and child?

- Provide a sympathetic ear
- Explanation about a possible explanatory model
  - Does that explanation link to the possible cause?
  - 'Medical' label (e.g. irritable bowel syndrome) or holistic approach ('from the heart')
  - In case of medical label: why? Do you believe parents expect a biomedical label from you?
- Rule out serious organic illnesses
  - Extensive medical history and physical examination
  - Diagnostic testing: **fecal calprotectin** / blood testing

III Treatment

- What treatment is effective for these children, according to you?
  - Does the treatment link to the cause?
  - Only patient education / refer for psychological help/ medication (e.g. laxatives)
- How do you find out the treatment is effective?
  - Do you plan a follow-up consultation? Or does the patient never return?
  - In case of no subsequent consultation: how do you know the symptoms have disappeared? Do you trust on the self-reliance of parents and child?

IV Diagnostic testing

- What are reasons to conduct or refrain from diagnostic testing?
  - Reassure parents and child
  - Diagnostic uncertainty or gut feeling
  - Rule out specific organic diseases
  - Alarming or persisting symptoms

IV Referral

- What are reasons to refer or refrain from referring to a medical specialist?
  - Parental request / diagnostic uncertainty / seek for specialized treatment of functional symptoms / 'wanting to do something '

V How does the FCal guided referral strategy help you in making management decisios?

- Reassure parents and child (to agree that there is no serious illness)
- To rule out IBD

- To refer less
- To use fewer blood tests
- Other:

In case it does not help, why not? Do other factors play a larger role (parental concern, persisting symptoms, daily limitation because of the symptoms)

Is there added value of using fecal calprotectin as a point-of-care-test instead of standard laboratory testing?

VI Online training about FCal guided referral strategy. What did you learn?

- Epidemiology different causes
- Disadvantages of referral to specialist care (medicalization, overdiagnosis, costs)
- Diagnostic characteristics fecal calprotectin (high sensitivity, better than blood tests)
- Indications and contra-indications fecal calprotectin (age 4-17 years, only in case of alarm symptoms for IBD)
- Interpretation and cut-off values (<50, 50-250 en >250)
- Communication functional gastrointestinal disorders (explanatory models, follow-up consultation)

STOP RECORDING

Appendix 4. Member check 2

GP 1 'I agree'

GP 4 'I do not understand this quote in this context?'

After making the following changes (in yellow and ~~strikethrough~~), GP 4 agreed with the use of the quote in the context:

Feeling capable to manage FGID in primary care – Many GPs believed that reassuring and labeling the symptoms (e.g. 'irritable bowel syndrome') is sufficient for parents and children to deal with the symptoms by themselves. In the GPs' experience, such a label was often enough to stop them from consulting the GP again and to deal with the symptoms by themselves. ~~When questioned whether the lack of consultations implies the absence of symptoms in children, GPs highlighted the inherent limitations of their role, emphasizing that they cannot resolve every issue.~~

> [about labeling] GP 3: *"That is also more efficient; then you can wrap it up … you can move forward."*
> GP 4: *"Yeah, and what [GP 6] just said ... it works well. I see those children very infrequently after. You've given them something to work with … of course, they still have their abdominal pain. If they come for something else, and you ask about it, they still have it occasionally, but it's not a problem they come back with often."*

When questioned whether the lack of consultations implies the absence of symptoms in children, GPs highlighted the inherent limitations of their role, emphasizing that they cannot resolve every issue.

GP 5 'My sentences don't flow very smoothly in the report. I'd rather phrase it like this: 'I don't feel insecure when assessing a sick child. The first look is the most crucial. There's already a lot of information to gather from thatt. So, I'm not quick to fear missing something in this regard.'

After responding with the following, GP 5 agreed with the use of the quote in the context and did not wish to make any further changes.

'Your new quote is indeed more elegantly formulated, but I believe its essence is similar to the original quote. Quotes are expected to have a natural flow, reflecting their origin from real spoken language. While I cannot modify quotes myself (as it would lack transparency), I can adjust the context or explanation. Currently, the context of the quote is as follows:

GPs expressed confidence during the diagnostic process for most children with chronic abdominal symptoms. They emphasized that the majority of these children have functional gastrointestinal disorders (FGID), allowing them to use tools other than diagnostic testing (e.g., growth monitoring, watchful waiting) to differentiate between children with FGID and those with serious organic illnesses.

"But I often don't feel insecure. Actually, when I see the child, I often have a sense of "this is fine." And, if you monitor them, you can see how things develop. No, I'm not worried about missing something." (GP 5)

Would you like me to add or modify anything here?'

GP 9 ***'Yes I agree with the content'***

GP 17 ***'I identify strongly with them. I fully endorse the quote. As a general practitioner, we can never completely rule out serious matters no matter how hard we try; unfortunately, you come to realize this when you've been in this profession for a long time.'***

GPs 2,3,6,7,8,10,11,12,13,14,15 and 16 did not provide a response to the member check.
